# Supplementary material for: Entropic associative memory for manuscript symbols
Source: PLoS One. 2022 Aug 4;17(8):e0272386. doi: 10.1371/journal.pone.0272386 (PMC9352019; doi:10.1371/journal.pone.0272386)
Supplement: Attachment — Submitted filename: Report.pdf [file pone.0272386.s001.pdf]

Report

The authors investigated the entropic associative memory for manuscript symbols. This topic is interesting and shall be worth studying. My comments are as follows:

1. The motivation of this work shall be improved.

2. What is the novelty of this article? What is the technical difficulty on methods and analysis aspect? Please give some explanations.

3. The practical application of entropic associative memory for manuscript symbols shall be presented in Conclusion.

4. Compared with the previous publications, what is the advantage of this paper?

5. The format of the references shall be united.

6. The following related publications on associative memory shall be added. e.g.,

1)Further investigation on bifurcation and their control of fractional-order BAM neural networks involving four neurons and multiple delays, Mathematical Methods in the Applied Sciences, 2021, https://doi.org/10.1002/mma.7581

2)6)Further analysis on dynamical properties of fractional-order bidirectional associative memory neural networks involving double delays, Mathematical Methods in the Applied Sciences, 2022, doi: 10.1002/mma.8477

3) Theoretical analysis and computer simulations of a fractional order bank data model incorporating two unequal time delays, Expert Systems with Applications 199 (2022) 116859.

4)Bifurcation properties for fractional order delayed BAM neural networks, Cognitive Computation 13(2) (2021)322-356.

5)New exploration on bifurcation for fractional-order quaternion-valued neural networks involving leakage delays, Cognitive Neurodynamics (2022) doi: https://doi.org/ 10.1007/s11571-021-09763-1

6)Bifurcation study for fractional-order three-layer neural networks involving four time delays, Cognitive Computation 14(2022)714-732.

7)Impact of leakage delay on bifurcation in fractional-order complex-valued neural networks, Chaos, Solitons & Fractals 142 (2021) 110535.
